# Parallelized 61‐Sample Inhibition Screening Accelerated by ^19^F‐MRI Compressed Sensing

**DOI:** 10.1002/smtd.71004

**Published:** 2026-09-06

**Authors:** Dilara Faderl, Alvar D. Gossert, Jan G. Korvink, Neil MacKinnon

**Affiliations:** ^1^ Institute of Microstructure Technology Karlsruhe Institute of Technology Eggenstein‐Leopoldshafen Germany; ^2^ Department of Biology ETH Zurich Zürich Switzerland

**Keywords:** ^19^F‐MRI, compressed sensing, parallelized hit screening, *T*_2_‐weighted imaging, trypsin inhibition

## Abstract

^19^F NMR is a well‐established technique for screening ligands in early drug discovery. However, conventional NMR measurements are inherently sequential, which limits experimental throughput. We recently demonstrated that ^19^F MR imaging (MRI) enables spatial parallelization of such measurements. In this study, we leverage the reporter assay format, where the transverse relaxation ($T_{2}$) of a fluorinated reporter ligand is monitored in the presence of potential competitors. Using $T_{2}$‐weighted MR images, up to 61 miniaturized samples can be resolved simultaneously. The screening assay operates at microliter‐scale volumes in a custom miniaturized honeycomb sample holder, providing a readily accessible and scalable quantitative binding assay. To further accelerate data acquisition, we developed optimized imaging protocols based on compressed sensing (CS), enabling substantial reductions in measurement time without compromising quantitative accuracy. This approach allows parallel screening of 55 potential ligands and efficient determination of relative binding affinities through simultaneous dose‐response experiments. We demonstrate the method on the model protein trypsin and the antibiotic target PPAT, characterizing several ligands with distinct binding properties. This study highlights the potential of MRI to enhance throughput and efficiency in early‐stage drug discovery markedly.

## Graphical abstract

One scan, swift answers: a ^19^F‐MRI honeycomb captures reporter ligand displacement across dozens of non‐fluorinated drug candidates in parallel. Miniaturized arrays with compressed sensing speed up acquisition, delivering rapid, quantitative readouts for ligand screening.

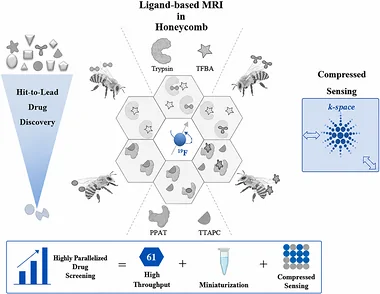

## 1 Introduction

The early drug discovery process involves several steps from first identification of hits to the development of lead compounds [1]. In the first step, compounds are screened against a target biomolecule to find initial hits that typically only have weak affinity. In a second step, the affinity of these ligands is quantified. The ligands with the highest affinity are modified in order to improve their affinity against the target, and later, lead compounds are developed where several other properties are optimized. During this early stage of drug discovery, typically, biophysical methods and biochemical assays are employed. The main requirements for these methods are: (i) high sensitivity to binding events, in order to detect weak primary ligands, (ii) high throughput to enable testing of as many potential ligands as possible, as hit rates are typically low, and (iii) the capability of determining the affinity of ligands, since this is the most important parameter to prioritize compounds in the pre‐clinical stages.

Several biophysical methods have been established like surface plasmon resonance (SPR) [2, 3], isothermal calorimetry (ITC) [4, 5], differential scanning fluorometry (DSF) [6], nuclear magnetic resonance (NMR) [7, 8], and others, which can be used both for initial hit identification and for subsequent affinity determination [9]. Hardly any of these methods fulfil all three criteria mentioned above, be it due to lack of sensitivity to weak binders (SPR, and enzymatic assays), low throughput (ITC), or unclear affinity determination (DSF, X‐ray). This typically makes it necessary to combine several assays [10, 11] which often yield conflicting results as the experimental conditions are not identical in the different approaches. NMR stands out in that it fulfills essentially all requirements: it has the highest sensitivity to weak binding events and is the preferred method for screening of fragment libraries with hits of very low affinity. NMR enables quantification of ligand–protein affinities over a wide dynamic range. Direct binding experiments provide dissociation constants ($K_{D}$) from the millimolar to low micromolar range, while competition assays extend the sensitivity to low nanomolar affinities [12, 13, 14, 15, 16, 17]. The apparent dissociation constant of the competitor ligand determined from the competition assay is denoted as $K_{C}$ [18, 19, 20].

However, the throughput of NMR experiments is generally relatively low. This limitation is less pronounced in initial screening, where thousands of compounds can be tested in mixtures. The bottleneck becomes more significant in subsequent optimization rounds, where individual compounds need to be tested for binding, and affinities must be determined in lengthy titrations. The low throughput is due to the inherently sequential nature of NMR measurements, where each sample is measured one at a time. Inspired by earlier work [21], we introduced a parallel format which takes advantage of magnetic resonance imaging to resolve a bundle of nine NMR tubes inserted at the same time into an MRI instrument [22]. Thus, the throughput was increased 9‐fold.

These measurements exploited the well‐established approach of (^19^F) ligand‐based screening in an indirect competitive mode. In this format, a fluorinated reporter molecule with low‐binding affinity is displaced by a non‐fluorinated competitor [14, 23]. The reporter's ^19^F signal is maximal in its free state, yielding bright image contrast in an MR image. Binding events reduce signal intensity (SI), producing grayscale levels quantitatively linked to the transverse relaxation time of the ^19^F reporter. Given the sensitivity of ^19^F transverse relaxation ($T_{2}$) to changes in the chemical environment, this technique enables the quantification of binding affinities of unknown non‐fluorinated competitors by measuring image contrast alterations resulting from reporter ligand displacement.

Here, we present a significant step forward by introducing a miniaturized sample holder, which allows the parallelized measurement of 61 samples, using only a fraction of the material that was required in the original format. We exploit an innovative ^19^F‐MRI reporter format to triage compounds for binding to different target proteins in a first step. For identified binders, the affinity is then determined in a parallel titration against the same fluorinated reporter. The capacity of the current sample holder thus allows testing 58 compounds for binding in parallel and determining the affinity of 10 ligands. However, the miniaturized format with cell diameters of 1.5mm, presents a significant challenge to MRI, since $T_{2}$‐weighted ^19^F MR images with high resolution need to be recorded efficiently.

In conventional MRI, full *k*‐space sampling is typically employed to preserve signal integrity, suppress image artifacts, etc. However, this requires a large amount of measurement time, and in high‐throughput MR screening applications focusing on ligand‐binding assays, the objective is simple estimation of $T_{2}$ rather than high‐resolution imaging; thus, exhaustive sampling may not be necessary. Accurate detection of binding events can be achieved using substantially undersampled data by exploiting signal sparsity in an appropriate transform domain (e.g., the wavelet domain). This leads to a more focused question: *What is the minimum k‐space sampling required to ensure reliable quantification and classification in sparsity‐driven magnetic resonance imaging assays?*

Compressed sensing (CS) is a widely adopted sparsity‐promoting method that facilitates undersampling by exploiting low *k*‐t sampling rates (sub‐Whittaker‐Nyquist‐Shannon). Using Cartesian *k*‐space trajectories with random undersampling patterns, the frequency‐encoding (FE) direction remains fully sampled, while only a subset of the phase‐encoding (PE) lines is acquired. The number of acquired PE lines scales linearly with the total acquisition time, thus shortening scan time. By acquiring only a fraction of the data, imaging time can be accelerated (e.g., 8‐fold acceleration when 32 out of 256 lines are acquired).

Assuming a dataset of N samples, a fully sampled acquisition requires approximately 2mN measurements according to the Whittaker–Nyquist–Shannon sampling criterion [24, 25], where m denotes the maximum spatial frequency. In the context of *k*‐space acquisition, let s denote the number of acquired complex‐valued *k*‐space samples. CS intentionally acquires only a subset of these measurements (s<2mN), thereby reducing the acquisition burden. Although this undersampling violates the Whittaker–Nyquist–Shannon sampling criterion required for conventional Fourier reconstruction and would therefore lead to aliasing artifacts, accurate image reconstruction remains possible by exploiting the sparsity of MR images together with incoherent *k*‐space sampling and iterative nonlinear reconstruction algorithms. Consequently, CS enables substantial reductions in acquisition time while preserving image fidelity and improving signal‐to‐noise ratio (SNR) [26].

Predicting the extent of acceleration achievable without compromising image quality and diagnostic efficacy is ambitious. CS techniques are effective when the necessary data points and their *k*‐space locations are known to ensure clinical relevance. Given that medical images are highly interpretable in specific transform domains and can be represented sparsely, CS is frequently utilized in medical imaging applications (e.g., 3D abdominal MRI, free‐breathing DCE‐MRI, 4‐D flow imaging) [27]. Hence the question arises: *Why not take advantage of compressed sensing for imaging‐based drug discovery as well?*

Here, we report a highly parallel and sparsely sampled hit‐to‐lead compound screening assay using a ^19^F‐MRI methodology. The assay is based on a transverse‐relaxation approach combined with CS undersampling to accelerate imaging time. By exploiting CS, we were able to reduce the imaging time from 18 to 2min per sample [22], while maintaining microliter‐scale sample volumes. By monitoring ^19^F‐signal alterations of the fluorinated reporter molecule 4‐trifluoromethyl‐benzylamine (TFBA), in competitive binding with 55 non‐fluorinated drug candidates arranged in a densely packed honeycomb array, we successfully screened potential inhibitors within a single ^19^F‐MR image acquisition. From this, 13 primary hits capable of displacing bound TFBA trypsin were identified. We then quantified their binding affinities ($K_{C}$) by measuring changes in $R_{2}$ relaxation times in a dose‐dependent manner.

To validate and further characterize weak binders, we constructed a reference library of known TFBA competitors with different binding affinities and compared their binding regimes.

As a disease‐relevant case study, we applied our screening workflow to the enzyme phosphopantetheine adenylyltransferase (PPAT). PPAT is an essential enzyme in bacterial synthesis of co‐factor A, rendering it a promising target in the development of antibacterial agents [28, 29]. By analyzing the displacement of the fluorinated reporter molecule (1‐[3‐(trifluoromethyl)‐[1,2,4]‐triazolo[4,3‐b]pyridazin‐6‐yl]piperi‐dine‐4‐carboxylic acid (TTAPC), we identified three non‐fluorinated inhibitors with differing binding strengths as competitive displacers. This demonstrates the successful extension of our ^19^F‐$T_{2}$ relaxation‐based drug screening methodology from the well‐characterized enzyme trypsin to the more complex, disease‐relevant enzyme PPAT.

## 2 Results and Discussion

### 2.1 Higher Sample Parallelization and Sparse Sampling

Recently, we demonstrated a ^19^F‐MRI ligand‐based assay using $T_{2}$ mapping to screen 9 samples containing ligand‐protein mixtures in parallel. Binding was determined in competition mode using the well‐studied trypsin‐benzamidine (BZD) system [22]. In the present work, sample parallelization and miniaturization were increased in conjunction with optimized MRI techniques for ligand screening, aiming to achieve the highest throughput while balancing SNR, resolution, and maximum acceleration factor (AC).

For the first step towards higher parallelization, the number of samples was extended from 9 (in the original work) to 61 using a custom sample holder in a honeycomb shape designed to exploit the accessible physical space inside our home‐built ^19^F‐coil [22].

To achieve optimal imaging resolution to ensure spatial discrimination of individual compartments (cells) within the custom‐built honeycomb sample holder (shown in Figure S1), in‐plane resolution was set to 64 × 64 while keeping the field of view (FOV) constant at 20mm. Within a 10mm image slice, $0.97\;mm^{3}$ was sufficient to visually distinguish between adjacent cells with a wall thickness 150μm (Figure 3C) at a reasonable measurement time.

**Figure 1:**
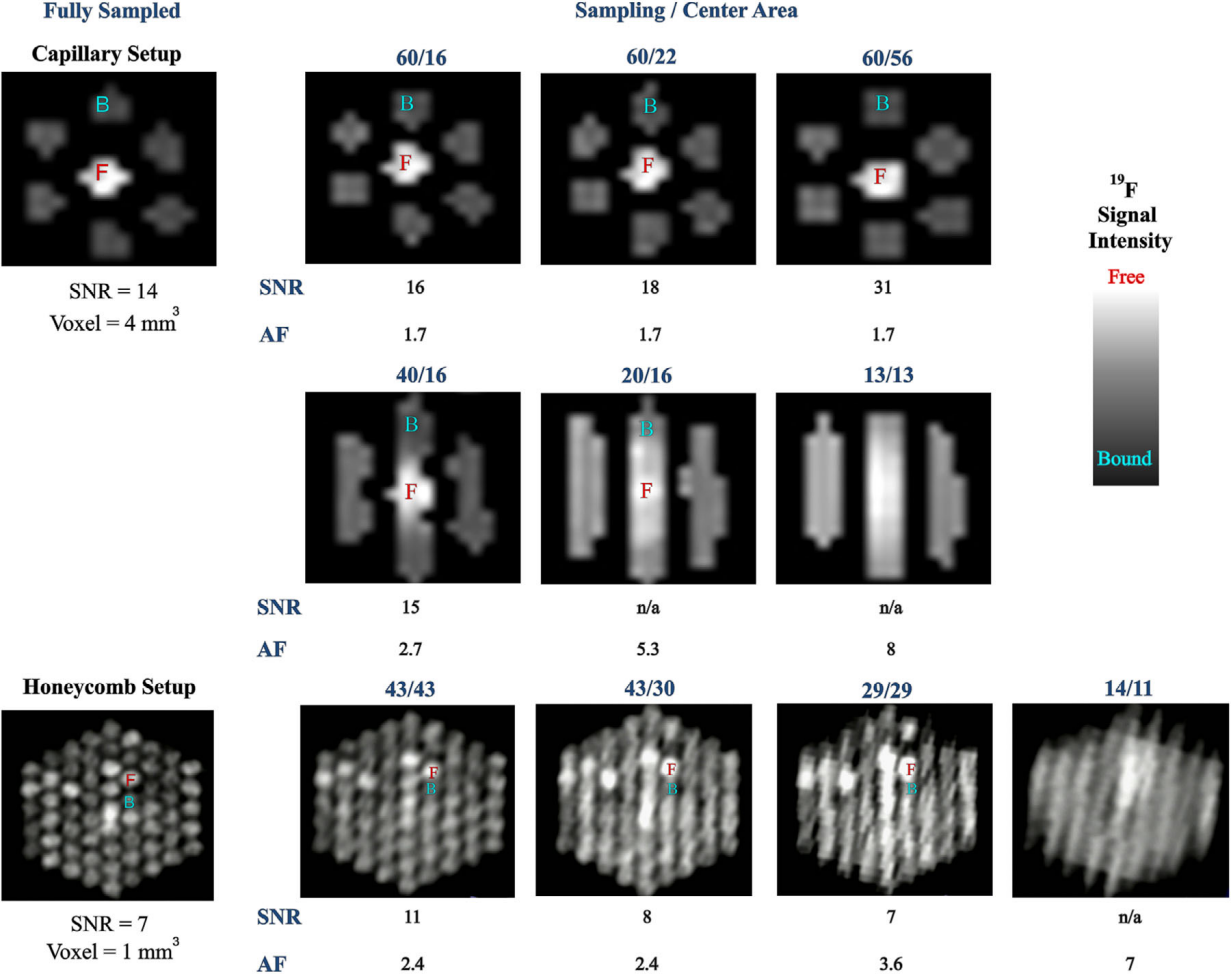
CS parameter optimization on capillary and honeycomb sample holder system using spin echo pulse sequence. ^19^F‐$T_{2}$‐maps of seven trypsin‐TFBA‐BZD experiments in capillaries and a 55 compound screen in a honeycomb sample holder are shown. Fully sampled (left column) and undersampled experiments with SNR values are given for comparison. *k*‐space sampling percentages and CA amounts were varied and are given over each image. AF values together with SNR are given beneath the images. Only two samples are labeled: F (red) = free TFBA, and B (cyan) = trypsin‐bound TFBA. The imaging parameters for the capillary system reference image were FOV 20mm×20mm, MTX 32×32, SL 10mm, TR/TE = 2.5s / 200ms, NEX 20, TA 38min and for the honeycomb sample holder FOV 20mm×20mm, MTX 64×64, SL 10mm, TR/TE = 6s / 200ms, NEX 500, TA 2h. n/a indicates conditions for which the parameter was not applicable.

**Figure 2:**
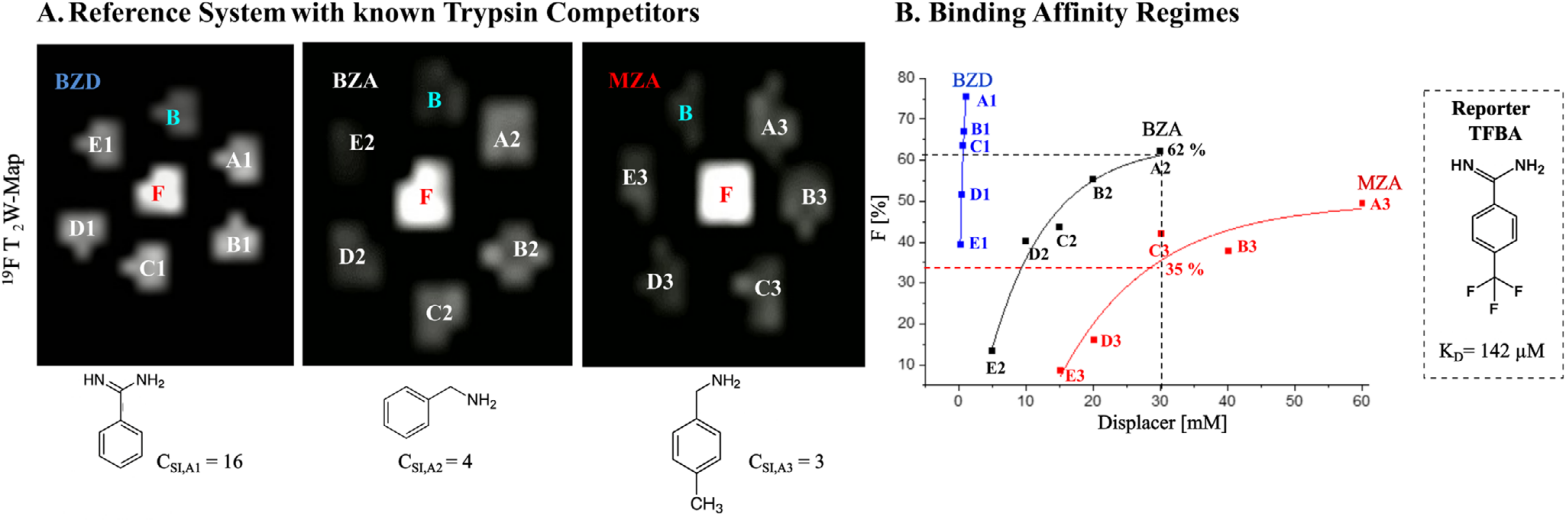
Reference competitors for TFBA displacement with BZD, BZA, MZA. (A) $T_{2}W$ snapshots of TFBA using fully encoded MSME imaging protocol, competitors at different concentrations (A–F). The competitor molecular structures and calculated signal contrast (calculated between sample A and F for each competitor) are given under the snapshots. (B) Displacement percentages (F) of bound TFBA (green) to trypsin (300 μM) are shown as a function of competitor concentrations in a range of (5 – 60 mM (white)). The TFBA displacement experiments (A–F) are in different concentrations: A indicates the highest competitor concentration and E the lowest. The numbers of experiments correspond to 1 for BZA and 2 for MZA, accordingly. The imaging parameters were FOV 20mm×20mm, MTX 32×32, SL 10mm, TE = 200ms, NEX 500, TA 2 h–4 h depending on the TR selection. Voxels correspond to a volume of approx. $4\;mm^{3}$ per sample. CS was not applied.

**Figure 3:**
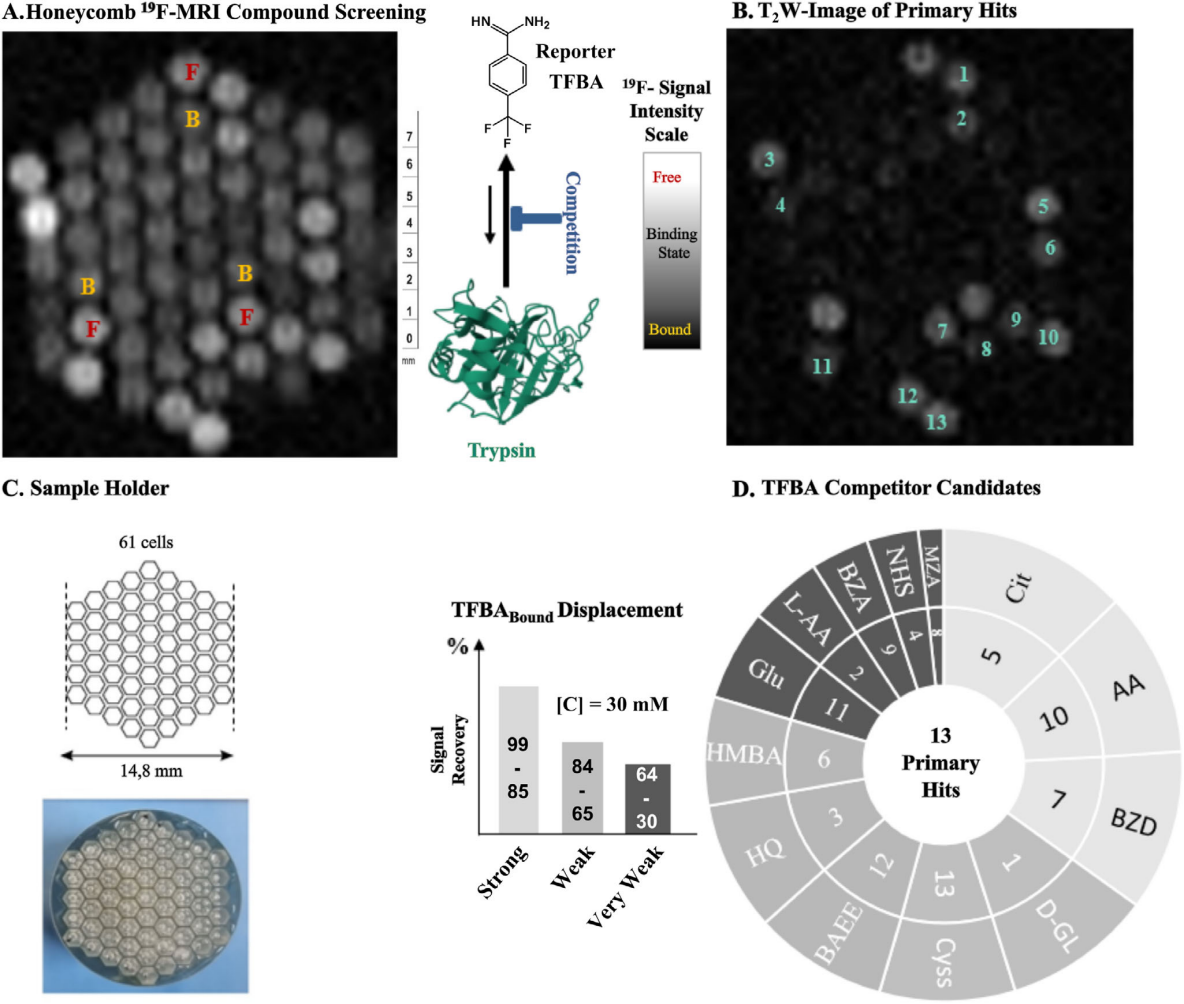
Miniaturized $T_{2}$‐weighted‐^19^F‐MRI compound screening in a honeycomb‐shaped sample holder accommodating 61 cells. (A) Localization of individual reference samples with ^19^F‐experiment with TE = 20ms: 5 mM free TFBA (F‐red), 5 mM TFBA in presence of 300 μM trypsin (orange‐B), partially displaced TFBA (other cells). (B) Detection of primary hits (30 mM (green)) upon displacement based on the ^19^F‐signal recovery of the trypsin‐bound TFBA using a spin echo RARE protocol. (C) Technical details of the honeycomb sample holder and a representative picture of one of the fabricated holders. (D) Validation of primary hits as competitor candidates based on their displacement strength at TE = 200ms. All listed chemicals with abbreviations are detailed in Tables 1 and 2. The imaging parameters were FOV 20mm×20mm, MTX 64×64, SL 10mm, TR = 2.5s, NEX 500, TA 2h 5min. Voxels correspond to a volume of $0.97\;mm^{3}$ per sample. CS parameters are 43% sampling / 43% CA. Structure visualization of trypsin (PDB ID: 1TGB).

Next, the reporter TFBA concentration was adjusted from 25 mM to 5 mM. By lowering the reporter concentration, we reduced the threshold for competition for the binding site of trypsin, sensitizing the assay to weak binders that could then displace TFBA (see Supporting Information for a TFBA concentration optimization for weak binders).

The optimal target (i.e., protein) concentration and the echo delay time (TE) for the spin echo RARE protocol were determined by investigating the ^19^F‐signal contrast $C_{\text{SI}}$ on the MR images using the honeycomb sample holder. By comparing the ^19^F SI of the TFBA reporter in the free (*f, free*) and trypsin‐bound states (*b, bound*), we calculated the contrast (adapted [20]):

(1) $$C_{SI}=\frac{SI_{f}-SI_{b}}{SI_{f}}\times 100$$

With 5 mM TFBA, a ^19^F‐$C_{\text{SI}}$ of 84% was achieved with a trypsin concentration 300 μM and 200ms as the TE time, which is optimal to ensure a large assay window for competition experiments where the contrast should be greater than 70% [20]. These values were used for single‐echo displacement experiments in all future experiments unless otherwise described.

To accommodate the increased sample density in this work, experimental parameters were updated relative to our previous 9‐capillary version [22]:

- The reporter ligand TFBA concentration and sample volume were reduced from 25 to 5 mM and from 200 to 50μL, respectively.
- Acquisition matrix size was increased from 32 × 32 to 64 × 64.

These modifications, particularly the 20‐fold reduction in the total amount of reporter ligand per sample (resulting from a 5‐fold reduction in TFBA concentration and a 4‐fold reduction in sample volume), required longer acquisition times to maintain sufficient SNR. In our previous study [22], a conventional single‐sample ^19^F $T_{2}$ measurement performed in a standard 5mm NMR tube (500μL) required approximately 18min per sample. Physical sample parallelization using the 9‐capillary holder reduced the effective acquisition time to approximately 6min per sample while maintaining comparable SNR conditions (SNR ≈ 15). In the present work, the honeycomb holder extends this concept by enabling the simultaneous measurement of 61 samples within a single MRI acquisition. The combination of high‐density sample parallelization provided by the honeycomb holder and CS reduced the effective acquisition time to approximately 2min per sample, corresponding to an overall 9‐fold acceleration compared with the conventional single‐sample experiment (see Table S3).

Since the CS technique is achieved by skipping some *k*‐space data that are normally acquired, two parameters in ParaVision (Bruker) were optimized while aiming to preserve quantitative accuracy of relaxation measurements and image quality: sampling (*S*) and center area (CA). The degree of sampling (*S*) altered the total number of PE steps collected relative to the whole *k*‐space. By adjusting the parameter CA, the distribution of collected *k*‐space data points between the central and peripheral regions was optimized. We investigated the optimal *k*‐space undersampling through CS parameter optimization, where image quality was kept as high as possible and extracted $T_{2}$‐relaxation time deviations were minimized compared to the fully encoded $T_{2}$‐weighted images (see Supporting Information, Compressed Sensing).

As a reference point, multi‐echo $T_{2}$‐relaxation experiments using CS were first carried out using the capillary system with a larger sample volume (200μL) to investigate the correlation between the two CS parameters, spatial resolution factor, and the $T_{2}$‐relaxation time of the observed protein‐ligand experiments. To accomplish this, seven capillaries containing 5 mM free TFBA with 300  μM trypsin, and five different BZD experiments ranging over 100 μM–500 μM were investigated by varying the acquired *k*‐space data sampling percentage and CA. In our CS $T_{2}$‐weighted‐^19^F‐MRI experiments, *k*‐space was acquired with the RARE encoding scheme (order of acquisition), where the PE lines are distributed across successive echo trains rather than being sampled strictly linearly.

The fraction of *k*‐space sampling was varied between 16% and 56% of the central area while keeping an overall sampling of 60%. The results were compared to the ${\mathrm{TFBA}}_{\mathrm{free}}$ sample from the fully sampled reference $T_{2}$‐weighted ^19^F‐MR image carrying 7 capillaries (sample “F” in Figure 1). The remaining noncentral *k*‐space data points were selected randomly in the outer spatial frequencies of the 32 x 32 matrix.

SNR analysis based on the sample denoted “F” demonstrated that an increased number in the central *k*‐space region by varying *CA* parameter only changed the SNR values proportionally without impacting the imaging time, since the low spatial frequencies located in this region encode image contrast and smooth structures. In the case of the honeycomb sample holder, less central *k*‐space data point collection led to aliasing distortions and caused signal overlapping of the neighboring samples. These artifacts were also observed when fewer PE steps (lower sampling points) were collected. Even though we lowered the *CA* values up to 16%, the blurring appeared only when the *S* was less than 50%, proving the inverse dependency for the Whittaker‐Nyquist‐Shannon criteria.

By varying *S* between 20% and 60% with a central *k*‐space coverage of 16% to investigate the trade‐off between *CA* and *S*, we aimed to identify the highest feasible AF. The $T_{2}$‐weighted images of sample F exhibited blurring artifacts as *S* decreased to 40%, and the derived $T_{2}$ relaxation times showed increasing deviations from 4ms for 56% *S* to 138ms when *S* fell below 30%. Although blurring effects were also present at 40% sampling, each experiment's region of interest (ROI) was moderately isolated to allow independent $T_{2}$‐time analysis, despite minor signal overlap between neighboring experiments. Thus, for both sample holder systems, we could reduce the *S* to 43 %–40 % with an AF of 2.7 for the capillary system and 2.4 for the honeycomb system and still perform distinctive ROI analysis on the undersampled ^19^F‐images (Figure 1). To eliminate signal overlap between neighboring cells for the honeycomb structure, the spatial resolution was enhanced by a factor of 4 compared to capillary system.

The optimal balance between SNR and acceleration was achieved with 43% coverage of the central *k*‐space and *S* of 43% for both sample holders. This configuration resulted in only a minor deviation (approximately 20ms) in the measured $T_{2}$ relaxation time for experiment F while enabling drug candidate screening per sample within just 2min. Therefore, these CS parameters were applied for the hit screening (phase 1) as well as for the binding affinity determination of the lead competitors (phase 2) described below.

### 2.2 Benchmarking With Known Competitors

Low‐affinity competitors may be mistakenly classified as non‐binders due to their minimal displacement effects, particularly in low signal contrast scenarios when acquiring MR images [20, 30, 31]. To mitigate this challenge and strengthen the reliability of our ^19^F‐MRI hit screening, we tested the limitations of this assay using a set of known trypsin competitors for TFBA spanning largely different binding affinities: BZD (strong binder, $K_{C}$ = 35 μM), BZA (weak binder, 300 μM), and MZA (very weak binder, $K_{C}$ = 1.5 mM) [32, 33, 34]. In these experiments, the capillary sample holder was used, as it accommodates a larger sample volume (200μL) compared to a single honeycomb cell (50μL), thereby improving SNR 4‐fold. The strategy was to determine the binding affinities of weak TFBA competitors, estimate detection thresholds for these competitors, and identify potential aggregation‐ or solubility‐related effects at high competitor ligand concentrations.

Fully encoded, dose‐dependent $T_{2}$‐weighted ^19^F‐MRI experiments were conducted using the same TFBA (5 mM) and trypsin (300 μM) concentrations to characterize the reference competitors in different concentration ranges (Figure 2).

^19^F‐signal‐based displacement analysis demonstrated detection sensitivity sufficient to distinguish the weak binder BZA and the very weak binder MZA, with a calculated $K_{C}$ of approximately 300 μM and 1.5 mM, respectively. The strongest competitor, BZD, achieved the highest displacement (75%) at 1 mM, whereas BZA and MZA induced maximum displacements of 60% at 30 mM and 50% at 60 mM, respectively. This pilot experiment established that affinities can be determined in titrations for compounds with affinities spanning two orders of magnitude and that even weak binders can be detected, provided the concentration is high enough. Based on the data in Figure 2B, a concentration of 30 mM was chosen for the displacement experiments for screening.

### 2.3 Highly Parallel Hit Screening

Once experimental conditions were optimized, a library of 55 compounds was screened to find primary hits that displaced bound TFBA from trypsin. We included triplicate control samples of free TFBA and trypsin‐bound TFBA for SI normalization, bringing the total number of samples to 61. Compound screening was done at 30 mM concentration, ensuring a large excess was available, such that also weak competitors would partially displace TFBA present at 5 mM concentration.

The screening experiment was performed using a RARE protocol with two specific TE times. The experiment with TE = 20ms served as a reference experiment. Notably, this experiment also determined the orientation of the honeycomb structure in the magnet. The distinct signals of positive (free TFBA, brightest image) and negative (bound TFBA, darkest image) controls were used to establish the cell locations of the controls, and the location of the competitor candidates was derived from the map of the filling pattern (Figure 3A).

Binders were identified using a two‐point Carr–Purcell–Meiboom–Gill (CPMG) experiment acquired at echo times of 20ms and 200ms, where $T_{2}$‐weighted contrast alterations were assessed. For binding analysis, only the images recorded at the longer echo time (TE = 200ms) were evaluated, as they provided binding‐induced alterations to $T_{2}$‐values reflected by the increased SI compared to the bound TFBA state. 13 primary hits from 55 compounds were identified based on the fraction of reporter ^19^F‐signal recovered (Figure 3B). The fraction of signal recovered was calculated by taking the ratio of the displaced ^19^F‐signal to the free TFBA ^19^F‐signal, where the displaced TFBA was the difference between the ^19^F‐signals in the displaced and bound states.

Hits were grouped into three affinity categories (strong, weak, very weak) according to their displacement relative to the reporter ligand TFBA, with thresholds set as follows: **strong** binders (F=99−85%, **weak** binders (F=84−65%, and **very weak** binders (F=64−30%). This classification guided further quantitative affinity determination (numerical details in Table S4).

The strongest displacement of the bound TFBA with a signal recovery of 99‐70% was caused by the hits with hydroxyl groups (1, 5, 10, and 13), and the known trypsin competitor 7. Hits 3, 6, and 12 caused a partial displacement above 50% and, therefore, were defined as weak binders. With the lowest signal recovery of 13%, hit **8** was categorized among other hits 2, 4, 9, and 11 as a very weak competitor (Figure 3D). The other 42 compounds were found to be non‐binders to trypsin.

Despite the acceleration that can be achieved, care must still be taken in interpreting the imaging data. The apparent variation in ^19^F‐SI likely originates from imaging‐related rather than chemical factors (e.g., compound 4 in Figure 3A,B). The detected ^19^F signal amplitude depends not only on molecular relaxation ($T_{2}$ decay) but also on slice‐selection geometry, coil sensitivity, and local susceptibility effects. In a multi‐cell honeycomb array, even small susceptibility artifacts (e.g., from bubbles or sample meniscus curvature) may yield signal intensities that do not reflect the chemistry. It is always useful to collect multiple slices along the sample wells to identify and exclude wells where such artifacts are found.

### 2.4 Binding Affinity Determination of Lead Competitors

In a second step, quantitative affinities of competitors from the selected 13 primary hits identified in screening were determined, using a dose‐dependent approach. This dose‐response format not only serves for quantifying affinity, but potentially, false‐positive hits can be eliminated, and other nonspecific high‐concentration effects can be assessed. For this purpose, $T_{2}$‐relaxation time alterations of the trypsin‐bound reporter TFBA ^19^F‐nuclei were monitored upon the addition of 13 hits in a titration series, using identical sample preparation and imaging parameters as before, except for the TE time window. A broader TE series ranging from 20ms to 1100ms was used to sample the full signal decay curve, allowing a more accurate and noise‐robust fit of the $T_{2}$ relaxation constant compared to the two‐point estimation in screening.

The hit concentration series was selected based on the TFBA ^19^F signal recovery profiles of primary hits classified as strong, weak, or very weak. BZD (hit 7) was used as a reference for a strong competitor, with a binding affinity of approximately 6 μM [22]. Accordingly, hits exhibiting signal recovery patterns comparable to hit 7 were measured at concentrations ranging from 500 μM to 10 mM. Given the pronounced strong displacement effect, concentrations exceeding 10 mM were not required. In contrast, very weak hits were evaluated at concentrations approaching their solubility limits, ranging from 20 mM to 60 mM (Figure 4).

**Figure 4:**
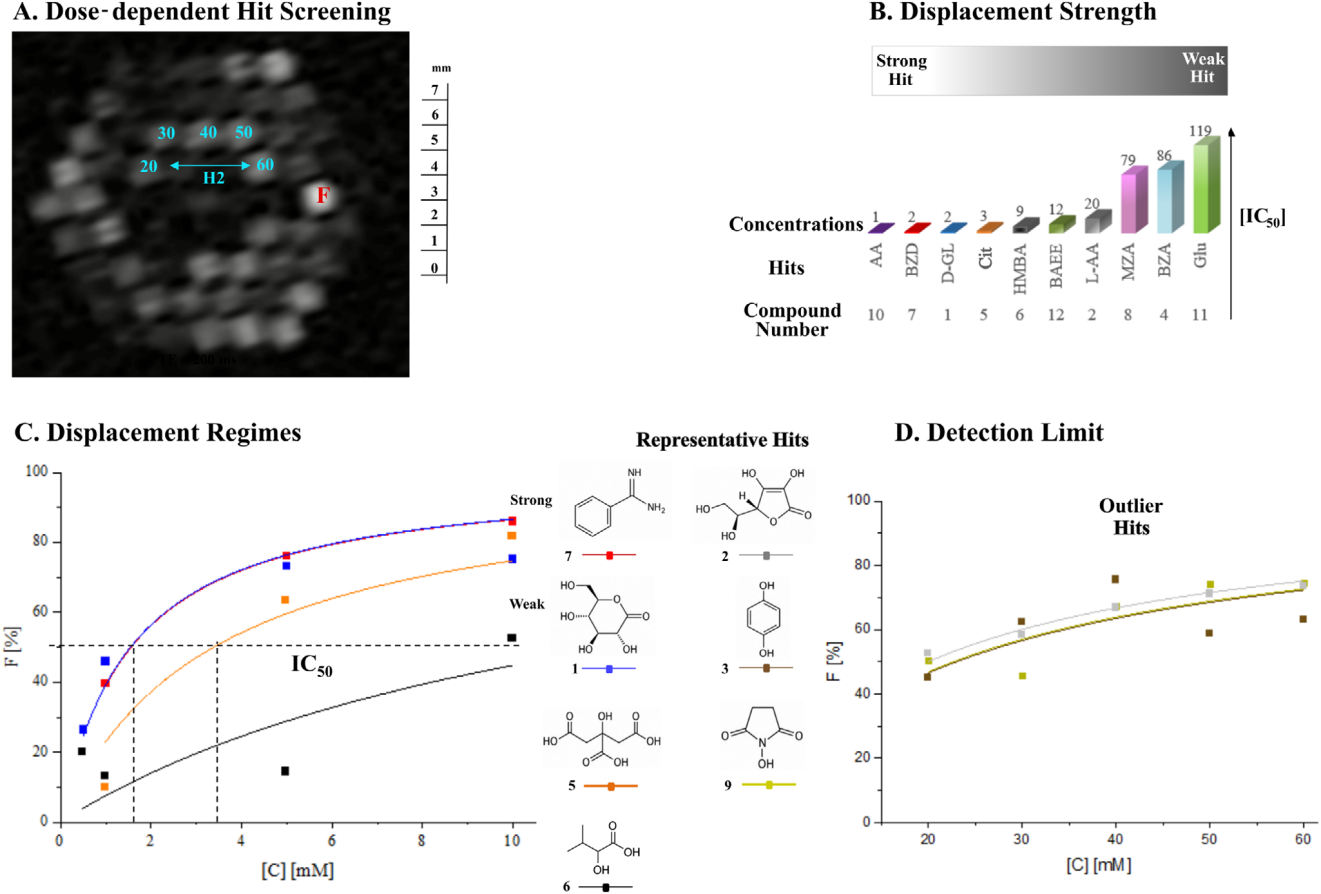
Competitor validation for trypsin based on TFBA displacement efficiency. (A) Impact of competitor concentration on ^19^F‐signal recovery of free TFBA, shown in a compressed‐sensed single $T_{2}$‐weighted image (TE = 200ms) of 13 primary hits. Honeycomb cell containing free TFBA is marked in red (F), and dose‐dependent displacement of the bound TFBA by hit 2 (H2, cyan). Numbers above the colored boxes indicate the competitor concentration in mM. (B) Ranking for primary hits, expressed as the competitor concentration ([C], mM) required to achieve 50% TFBA displacement ($IC_{50}$). Lower $IC_{50}$ values indicate stronger binding competitors. (C) Two displacement regimes (strong, weak) with representative competitors and their chemical structures (compounds 1‐9) plotted as a fraction of free TFBA signal recovery (F, %) versus competitor concentration. The dashed horizontal line marks the $IC_{50}$ threshold. Compound 7 served as a reference strong displacer. (D) Outlier displacement profiles (compounds 2, 3, and 8) showing upward curvature at high concentrations, indicating possible protein‐ligand aggregation. The imaging parameters were FOV 20mm×20mm, MTX 64×64, SL 10mm, TR/TE = 6s / 200ms, NEX 400, TA 2h. Voxels correspond to a volume of $0.97\;mm^{3}$ per sample. CS parameters are 43% sampling / 43% CA.

The displacement values (F, %) of the reporter ligand TFBA were extracted from the transverse relaxation rates ($R_{2}$), where F represents the percentage of reporter ligand displaced by the competitor. The $R_{2}$‐values were calculated from the measured $T_{2}$ relaxation times in three binding states: free state $\left(R_{2,f}\right)$, trypsin‐bound state in the absence of a competitor ligand $\left(R_{2,nc}\right)$, and TFBA displaced in the presence of trypsin and competitor ligand $\left(R_{2,c}\right)$ [18].

(2) $$F=100\times \left(1-\frac{R_{2,c}-R_{2,f}}{R_{2,nc}-R_{2,f}}\right)$$

Here, *F* = 0% indicates no displacement (fully bound reporter), and *F* = 100% represents complete displacement to the free state. The F values were fit with the four‐parameter competitor concentration dose‐response equation [35]:

(3) $$F=A-\frac{\left(A-A_{0}\right)}{1+{\left(\frac{\left[C\right]}{IC_{50}}\right)}^{n}}$$

where F represents the normalized displacement fraction corresponding to the baseline $A_{0}$ where there was no competing ligand present ([*C*] → 0) and *A* was set to 100 for maximum displacement indicating the full displacement of the bound TFBA. $IC_{50}$ is the extracted value after fitting the F values calculated for competing molecule C, and reflects the concentration required to displace half of the bound TFBA. The steepness of the curve, n (Hill coefficient), reflecting the cooperativity of the displacement process, was fixed at 1 during curve fitting, assuming a simple non‐cooperative one‐site competitive binding model. This assumption is required for the application of the classical Cheng–Prusoff relationship to convert $IC_{50}$ values into apparent dissociation constants ($K_{C}$) for the competitor ligands [36].

By extracting the $IC_{50}$‐parameter, we aimed to achieve rapid and robust estimation of competitive binding affinities, facilitating hit ranking and comparison across compound series.

Utilizing the $IC_{50}$‐values, we classified hits 1, 5, 7, and 10 as strong binders, defined as compounds with $[IC_{50}]<5\;mM$, since they require relatively low competitor concentrations to achieve 50% displacement of TFBA.

Hits 6, and 12, by contrast, exhibited $[IC_{50}]>5\;mM$ and were therefore considered weaker displacers (Figure 4B).

Hits 4, 8, and 11 showed the weakest displacement effects and their corresponding F‐values were near or outside the range covered by the selected concentration series (Figure 4B). Quantification of low‐level displacement (20 to 40% F‐values) requires a minimum signal contrast index ($C_{\text{SI}}$) of 5 according to the analysis of reference compounds. Therefore, for these hits, no reliable $IC_{50}$‐value could be determined, and these hits would probably be discarded for further development.

Since hits 4 and 8 were known weak competitors of trypsin, we performed additional displacement experiments with higher signal‐to‐noise. To this end, the capillary‐based system with a higher sample volume was used. In this way, their binding affinities were then determined accordingly and listed in Table 2.

**Table 1:** $K_{C}$ values for strong TFBA competitors obtained from the $IC_{50}$ values determined in dose‐dependent displacement experiments using the honeycomb sample holder. Note that the determined $K_{C}$‐values are below the protein concentration, where Equation (4) becomes less accurate.

Hit	Abbreviation	Chemical name	$K_{C}$ [mM]	Literature values [mM] or explanation
1	D‐GL	D‐(+)‐Glucono‐delta‐lactone	0.04±0.01	—
5	Cit	Citric acid	0.09±0.03	—
6	HMBA	2‐Hydroxy‐3‐methylbutyric acid	0.24±0.09	—
7	BZD	Benzamidine	0.05±0.01	0.013 [38] ‐ 0.016 [19] ‐ 0.035 [33]
10	AA	Alginic acid	0.04±0.02	—
12	BAEE	N‐benzoyl arginine ethyl ester	0.3±0.1	Substrate (0.25 < $K_{m}$ < 1) [39]

**Table 2:** $K_{C}$ of weak competitor candidates determined in the capillary setup via single‐concentration point with an exception of MZA and BZA via dose‐dependent $T_{2}$‐mapping.

Hit	Abbreviation	Chemical name	$K_{C}$ [mM]	Literature values [mM]
2	L‐AA	L‐Ascorbic acid	1.15±0.01	—
3	HQ	Hydroquinone	18±4	Nonbinder
4	BZA	Benzylamine	0.4±0.2	0.3 [33, 34]
8	MZA	Methylbenzylamine	1.75±0.01	1.5 [33]
9	NHS	N‐Hydroxysuccinimide	1.19±0.01	—
11	Glu	Glutamate	9±1	Nonbinder

The determined $IC_{50}$ values are highly useful for ranking individual hits; however, they only reflect the potency of a hit within a given assay. For example, in an assay with a 10‐fold lower reporter ligand concentration, lower competitor concentrations would result in 50% displacement. Therefore, to enable comparison between different assays, the apparent dissociation constant ($K_{C}$) of the competitor ligand was determined. Following previous studies [18], $K_{C}$ was derived from the competition binding assay using the known dissociation constant of the reporter ligand TFBA ($K_{D}=142\;\mu M$) [37]:

(4) $$K_{C}=\frac{\left(100-F\right)\left[C\right]K_{D}}{F(\left[L_{T}\right]+K_{D})}$$

where $L_{T}$ is the concentration of the reporter ligand (here TFBA). Rearrangement yields:

(5) $$K_{C}=\frac{\left[IC_{50}\right]K_{D}}{\left[L_{T}\right]+K_{D}}$$

The $K_{C}$ values of the competitor ligands ranged from 40 μM to 220 μM, corresponding to a broad spectrum of binding affinities, as listed in Table 1.

Hits 2, 3, 4, 9 and 1 were reassessed using the reference compounds (see 2.2 Figure 2A). The TFBA displacement of those competitors at a ligand concentration of 30 mM was compared with the F‐values of BZD (**7**), BZA (**4**), and MZA (**8**). We extracted the ^19^F‐value for reference competitors at the concentration of 30 mM: 62% for BZA and 35% for MZA (Figure 2B).

Ultimately, we categorized hits 3 and 11 as non‐binders since the ^19^F‐ signal recovery was less than 10% with a binding affinity of 10 mM–20 mM (Table 2). Hit 9 displaced the bound TFBA in a similar range as MZA, which was categorized as a very‐weak binder rather than a possible aggregation. For this very weak displacement regime, it was necessary to collect an image with a minimum signal contrast of 10%–15% between unbound and displaced free reporter ligand experiments with an SNR value of 3.

We confirmed that competitors can be ranked by combining two metrics: (1) dose‐dependent reporter ligand displacement and (2) $IC_{50}$ comparison with a reference competitor measured under identical sample conditions. These parameters were sufficient for $K_{C}$ determination, thus, can be obtained in parallel, enabling faster and more efficient screening.

### 2.5 Application to the Antimicrobial Target Protein PPAT

To test the ^19^F‐MR method in the miniaturized honeycomb format, we applied this approach to an actual drug target.: the enzyme PPAT, which is essential for bacterial synthesis of coenzyme A. Here, the displacement of the fluorinated reporter TTAPC (1‐[3‐(trifluoromethyl)‐[1,2,4]triazolo[4,3‐b]‐pyridazin‐6‐yl]‐piperidine‐4‐carboxylic acid) was monitored upon the addition of three known PPAT ligands (compounds 14, 15, 16), alongside six previously tested hits (1, 5, 6, 7, 10, 12), which served as controls.

Dose‐dependent $T_{2}$‐mapping of PPAT‐bound TTAPC was performed using the same reporter ligand concentration (5 mM) as for TFBA. However, the protein concentration was set to 25 μM, lower than in the trypsin study as ^19^F‐NMR experiments demonstrated sufficient contrast using a PPAT concentration within the range of 20–25 μM [31].

Utilizing the $T_{2}$‐filter (TE = 200ms), we quantified the competitive displacement of TTAPC by extracting dose‐dependent $R_{2}$ values for each compound and converting the resulting $IC_{50}$ values (Figure 5C) into apparent competition constants $K_{C}$ according to Equation (5). For this calculation, the experimentally determined dissociation constant of TTAPC ($K_{D}=440\;\mu M$) was applied. The transverse relaxation rates of TTAPC were determined to be $R_{2}=2.70\;s^{-1}$ for the free ligand and $R_{2}=14.29\;s^{-1}$ for the bound state, and these values were used consistently across all analyses. The resulting $K_{C}$ values are summarized in Table 3.

**Table 3:** $K_{C}$ of PPAT hits determined in the honeycomb setup via dose‐dependent $T_{2}$‐mapping.

Hit	Chemical name	$IC_{50}$ [mM]	$K_{C}$ [mM]
16	Methotrexate	0.22	18±5
15	Benzoaminomethylphenol	0.58	50±10
14	Biphenyl‐phenyl urea	6.16	498±8

**Figure 5:**
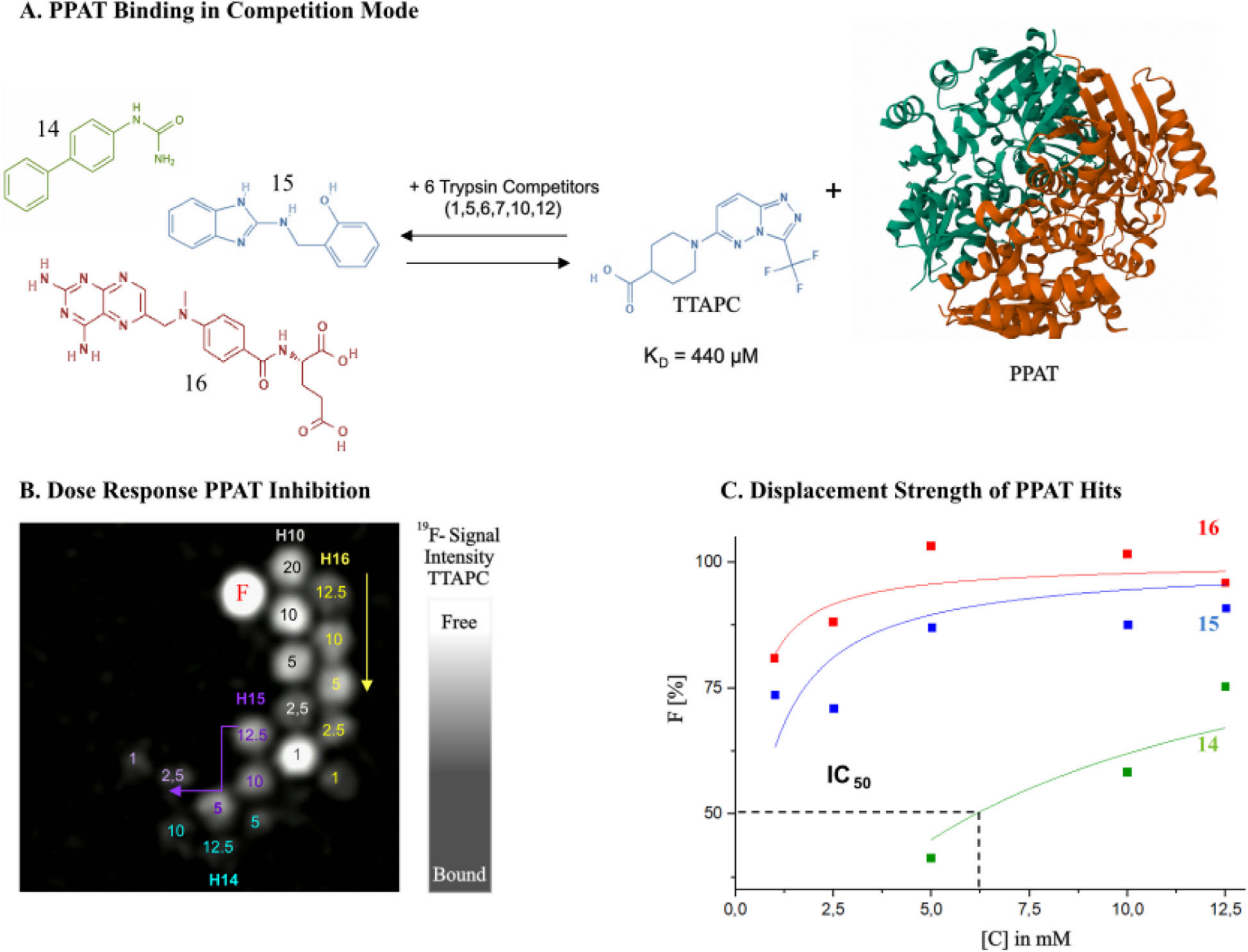
Dose‐dependent displacement monitoring for the PPAT reporter ligand TTAPC via $T_{2}W$‐^19^F‐mapping. (A) In addition to lead hits for trypsin, three known PPAT competitors (shown with the chemical structures; **14**‐ Biphenyl‐phenyl urea, **15**‐Benzoaminomethylphenol, and **16**‐Methotrexate) were screened for displacement. (B) A fully encoded dose‐response $T_{2}$‐mapping showed the ^19^F‐signal alterations of the bound TTAPC by filtering only the displacers using a TE time of 200ms (colored arrows show the direction of the concentration series). (C) Displacement strength of the hits using F‐values measured by $T_{2}$‐relaxation times with determined $IC_{50}$‐values. The imaging parameters were FOV 20mm×20mm, MTX 64×64, SL 10mm, TE/TR = 200ms/ 6s, NEX 200, TA 1h. Voxels correspond to a volume of $0.97\;mm^{3}$ per sample. CS parameters are 43% sampling / 43% CA. The PDB ID for the structural visualization of the PPAT enzyme is 6CCS.

Based on this analysis, hit 16 emerged as the strongest competitor, with an apparent $K_{C}$ = 18 μM. Hit 15 showed a substantially weaker affinity, with $K_{C}$= 47 μM, corresponding to an approximately 2.5‐fold lower competition efficiency relative to hit 16. Hit 14 produced only modest TTAPC displacement, yielding $K_{C}$ = 498 μM, nearly one order of magnitude weaker than hit 15 and more than 25‐fold weaker than hit 16.

Hit **10** was initially identified as binder, as it appeared to increase the TTAPC signal in the presence of PPAT, however, the dose‐dependent $T_{2}$‐values did not follow a systematic trend, indicating that no reproducible competitive displacement occurred. Therefore, the dose‐response experiment acted here as a important validation step to identify false positives from the initial screening. We attribute the erroneous behaviour of hit 10 to its poor solubility at the mM concentrations that were employed here; the precise solvent compositions and a visual documentation of hit 10 upon DMSO addition are provided in the Supporting Information.

In summary, these results highlight the utility of the ^19^F‐MRI approach for distinguishing competitors across a broad range of relative affinities, and equilibrium constants can be determined if the $K_{D}$ of the reporter ligand is known. Incorporating additional low‐concentration data points would further refine the displacement regression and provide a more complete characterization of the competitive profile. Nevertheless, this example illustrates how the method enables rapid, parallel prioritization of ligand candidates. Beyond the quantitative assessment of ligand binding, the method also proved sensitive to physicochemical irregularities in the samples such as aggregation or phase separation. Dose‐response curves and careful examination of the images allowed identifying such false positive hits. Collectively, this highlights the versatility of the ^19^F‐MRI workflow as both a binding‐affinity screening tool and a qualitative quality‐control readout for identifying sample‐related artifacts or formulation issues.

## 3 Conclusion

The pace of drug discovery is fundamentally limited by how rapidly compounds can be screened. Equally important, the versatility of the workflow allows interrogation of ligands across a broad affinity range while remaining compatible with microliter‐scale assay volumes used in pharmaceutical discovery. To advance our rapid ^19^F‐MRI‐based ligand screening methodology, we implemented an optimization step focused on increasing throughput, versatility, and MR image under‐sampling by leveraging built‐in instrument capabilities without requiring complex pulse programming or costly hardware modifications.

By customizing the sample holder from a bundle of 5 mm sample tubes to honeycomb, we could accommodate 61 physically isolated compartments in the same signal environment successfully imaged by using the maximum available physical space in the ^19^F‐coil. With each cell containing a separate ligand‐binding reaction, we simultaneously screened a library of 55 non‐fluorinated drug candidates for protein binding within a single measurement.

Using $T_{2}$‐mapping as the readout, we detect binding‐induced signal changes in the reporter ligands TFBA and TTAPC that yield key information: hit/no‐hit identification based on the $T_{2}$‐weighted contrast, competition fraction (%F), and determination of binding affinities ($K_{C}$) via $R_{2}$‐relaxation in dose‐response titrations.

In screening mode, we identified 13 hits for trypsin based on the recovered ^19^F signal of the reporter ligand TFBA using a single $T_{2}$‐filter (TE = 200ms). In hit characterization, we determined the binding affinities ($K_{C}$) of six lead compounds (**1**, **5**, **6**, **7**, **10**, **12**) for trypsin, and evaluated the $K_{C}$ values of three compounds (**14–16**) with a wide range of affinities for PPAT. Thereby, this screening approach was extended from the well‐characterized trypsin system to the disease‐relevant protein PPAT.

Strong displacers (**1, 5, 7, 10 for trypsin; 14, 15, 16 for PPAT**) were reliably detected within the honeycomb setup, with optimized conditions pushing sensitivity limits to sample volumes of 50μL at reporter concentrations of 5 mM. For weak binders (**2, 3, 4, 8, 9, 11**) we reached the quantitative detection limit of $R_{2}$‐based analysis, which was then overcome by integrating a capillary system that improved SNR.

The concentrations of reporter ligand and potential competitors are 2‐3 orders of magnitude higher than in conventional biochemical assays and NMR binding experiments. The reporter concentration of 5 mM, which is necessary due to the relatively low sensitivity of MRI, sets a high threshold for displacement, as potential competitors need to compete with the reporter at this concentration. This presents some challenges in terms of compound solubility and detection limits for weak binders. The high concentrations of 30 mM required in screening are above the solubility limit of many organic compounds, such that aggregation and associated artifacts can occur. We therefore expect that the full potential of the presented assay will be exploited when paired with hyperpolarization of the reporter ligand, which will allow reducing the concentrations of all components by orders of magnitude [40, 41, 42]. Nevertheless, with the current setup, we were able to efficiently identify ligands and characterize their affinities. This is due to the relatively low affinity of the employed reporter ligands, which could be displaced even by weak hits ($K_{C}$>1 mM). The tested competitors had high solubility, such that they could be employed at concentrations of 30 mM and above.

Implementing CS to the optimized high‐throughput pipeline, the measurement time was reduced through capturing only 43% of *k*‐space. With sufficient SNR (> 3) with minimal deviations in $T_{2}$‐relaxation times, even under violation of the Whittaker‐Nyquist‐Shannon criterion, we achieved robust compound screening in approximately 2h per full set, corresponding to 2min per sample. Imaging time was significantly reduced by a factor of 9‐fold (multi‐point $T_{2}$‐relaxation mapping approx 20min [43]) while still achieving high‐quality image contrast.

In case of utilizing different sample holder designs, we have shown the necessity of adapting the spatial resolution, in our case the voxel size changed from $4\;mm^{3}\;\;\;\mathrm{to}\;\;1\;mm^{3}$. More importantly, for the contrast‐quantified MRI protocols, it is important to find the trade‐off between the amount of the central *k*‐space and blurring artifacts on the images, which is followed by the next step of optimization in the total undersampling rate.

By combining miniaturized 61‐cell arrays with CS, we achieved a high‐throughput MRI rate and successfully optimized a streamlined workflow for competitive drug screening using ^19^F‐MRI, which can be readily adapted to ^1^H‐MRI. When paired with hyperpolarization techniques, this workflow has the potential to transform protein‐ligand detection into a rapid, parallel, and scalable platform, enabling high‐content molecular screening. This opens new possibilities for early‐stage drug discovery, fragment screening, and multiplexed assays.

While demonstrated on a 15.2T BioSpec MRI system with a custom‐built ^19^F RF probe, the proposed methodology of simultaneous detection is readily transferable to other MRI and NMR imaging [21] platforms supporting ^19^F detection and imaging gradients [44, 45]. At lower magnetic field strengths, the same approach remains applicable, with reduced SNR primarily compensated by longer acquisition times. The most likely trade‐off will be the degree of sample parallelization, with the honeycomb system requiring scaling to fit the appropriate RF detectors in the target system.

## 4 Experimental Section

*Honeycomb sample container*: The holder consists of 61 hexagonally arranged cylindrical cells, each with an internal diameter of 1mm and separated by 150μm thick walls. The diameter of the holder is 14.8mm, designed to fit within a 15mm ^19^F‐MRI coil. The sample holder was fabricated by 3D‐Druck‐Service (https://www.3d‐druck‐service.ch , Zurich, Switzerland. Accessed 10.07.2026), on a 3D Systems VIPER Si2 printer using Somos Prototherm 12120HT resin.

*Chemicals*: Trypsin from bovine pancreas was purchased as a lyophilized powder from Sigma‐Aldrich (T9935), and (trifluoromethyl)benzamidine (TFBA) was obtained from Enamine (EN300‐73109). The *E. coli* protein Phosphopantetheine adenylyltransferase (PPAT) was prepared as described by Lorz et al. [31]. Hydrophilic and hydrophobic competitor ligands were listed in the supplementary documentation.

*Characterization*: All ^19^F‐$T_{2}$ relaxation experiments were performed on a Bruker 650MHz (15.2T) BioSpec imaging system (Bruker BioSpin, Ettlingen) with a 15mm diameter home‐built ^19^F‐coil. Magnetic resonance (MR) images were acquired with RARE (Rapid Acquisition with Relaxation Enhancement) protocol and $T_{2}$‐mapping experiments were performed using a multi‐echo spin‐echo (CPMG‐type) sequence as implemented in ParaVision 360 (V 3.3). Imaging parameters are provided in the respective figures unless stated otherwise.

Representative results were validated across a minimum of three independent experiments. Statistical analyses were conducted using OriginPro 2025b (OriginLab Corporation, Wellesley Hills, USA).

## Conflicts of Interest

J.G.K. is a shareholder of the KIT spinout Voxalytic GmbH. The other authors declare no conflicts of interest.

## Supporting information


**Supporting File**: smtd71004‐sup‐0001‐SuppMat.pdf.

## Data Availability

The data that support the findings of this study are openly available in KITopen at https://www.bibliothek.kit.edu/english/kitopen.php , reference number 10.35097/j2ryda8x9m31qaea.
