# Supplementary material for: Parallelized 61‐Sample Inhibition Screening Accelerated by 19F‐MRI Compressed Sensing
Source: Small Methods. 2026 Sep 6;10(19):e71004. doi: 10.1002/smtd.71004 (PMC13646067; doi:10.1002/smtd.71004)
Supplement: Supplementary file 1 [file SMTD-10-e71004-s001.pdf]

Supporting Information:

Parallelized 61-sample inhibition screening accelerated by ^{19}F-MRI compressed sensing

Dilara Faderl,† Alvar D. Gossert,† Jan G. Korvink,*,† and Neil MacKinnon*,†

†*Department of Biology, ETH Zurich, Switzerland*

‡*Institute of Microstructure Technology, Karlsruhe Institute of Technology (KIT), Germany*

E-mail: jan.korvink@kit.edu; neil.mackinnon@kit.edu

Contents

1 Enginering **2**

2 Chemistry **2**

2.1 Solution preparation 2

Trypsin-TFBA 2

PPAT-TTAPC 3

Sample loading 3

3 Biophysics **3**

3.1 Compressed Sensing 3

3.2 k-space representation for CS 4

3.3 Python code for CS simulation 4

3.4 TFBA concentration optimization 6

3.5 Binding affinity classification for screening 6

3.6 PPAT - hit 10 R_2-relaxation rate 7

References **12**

1 Enginering

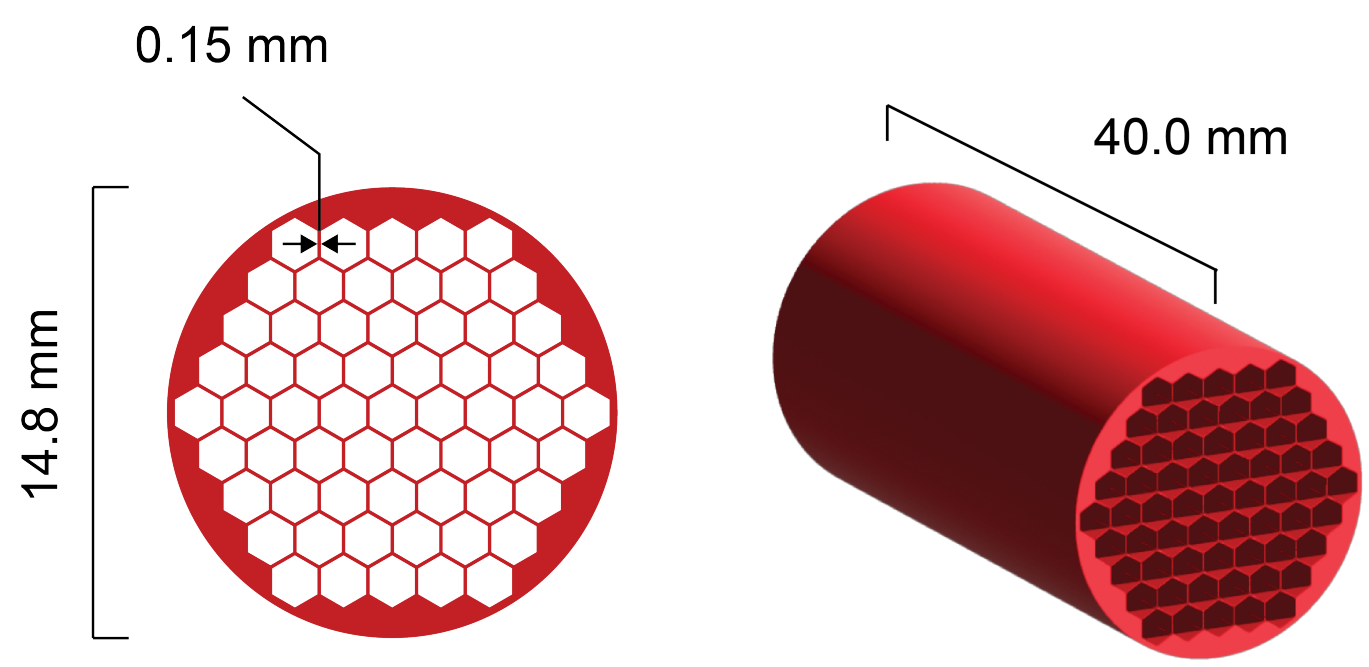

Figure S1: Technical drawing of the 3D-printed honeycomb sample holder with 61 cells of approximately 50 μl volume.

2 Chemistry

2.1 Solution preparation

In this study, we have prepared two different reporter ligand and protein solutions: Trypsin (protein 1) - TFBA (reporter ligand 1) and PPAT (protein 2) with TTAPC (reporter ligand 2).

Trypsin-TFBA

A ligand-protein stock solution of 50 mM of TFBA with 1.5 mM of trypsin was prepared in a PBS buffer (1x, pH 7.4, ThermoFisher, 10010-015), aliquoted, then frozen at $-20\,^{\circ}$C and stored until further use.

The hydrophilic competitors were prepared in PBS buffer (Table S2); meanwhile, hydrophobic ones were prepared in different solvents listed in Table S1. A 50 mM stock solution of those compounds was prepared for phase 1 and phase 2, aliquoted, frozen, and stored at $-20\,^{\circ}$C until further use.

PPAT-TTAPC

A solution containing PPAT and TTAPC was prepared by combining a 20 mM TTAPC stock solution (dissolved in 7:8 v/v DMSO:PBS) with a 100 µM PPAT stock solution, and diluting with PBS to obtain the final protein and reporter concentrations. Competitor compounds were added from two categories of stock solutions: (i) PPAT hits 14, 15, and 16 with a final solvent composition of **1:1.5 (v/v) DMSO:PBS**, and (ii) trypsin hits 1, 5, 6, 7, and 12 with a final solvent composition of **1:2.6 (v/v) DMSO:PBS**. In all cases, the protein, reporter ligand, and competitor ligands were combined such that the final PPAT and TTAPC concentrations remained constant across samples.

Sample loading

The compound mixtures for both protein systems were then filled into two different sample holders: 2.4 mm OD glass capillary (CM Scientific) or the honeycomb sample holder with a microliter syringe (Hamilton Central Europe S.R.L.) (shown in Section 1). Both ends of the sample holders were sealed by UV glue (Delo Photoband). All samples were freshly prepared on the measurement day and kept in a cooled transport container. They were then placed into a custom-built ^{19}F-coil for data acquisition (probe details in[S1]).

3 Biophysics

3.1 Compressed Sensing

The compressed Sensed (CS) images were reconstructed directly in ParaVision 360. In the reconstruction interface, the following parameters were used and kept the same for all CS experiments (Figure S2). Only CS parameters (sampling and center area percentage) were varied in the resolution card.

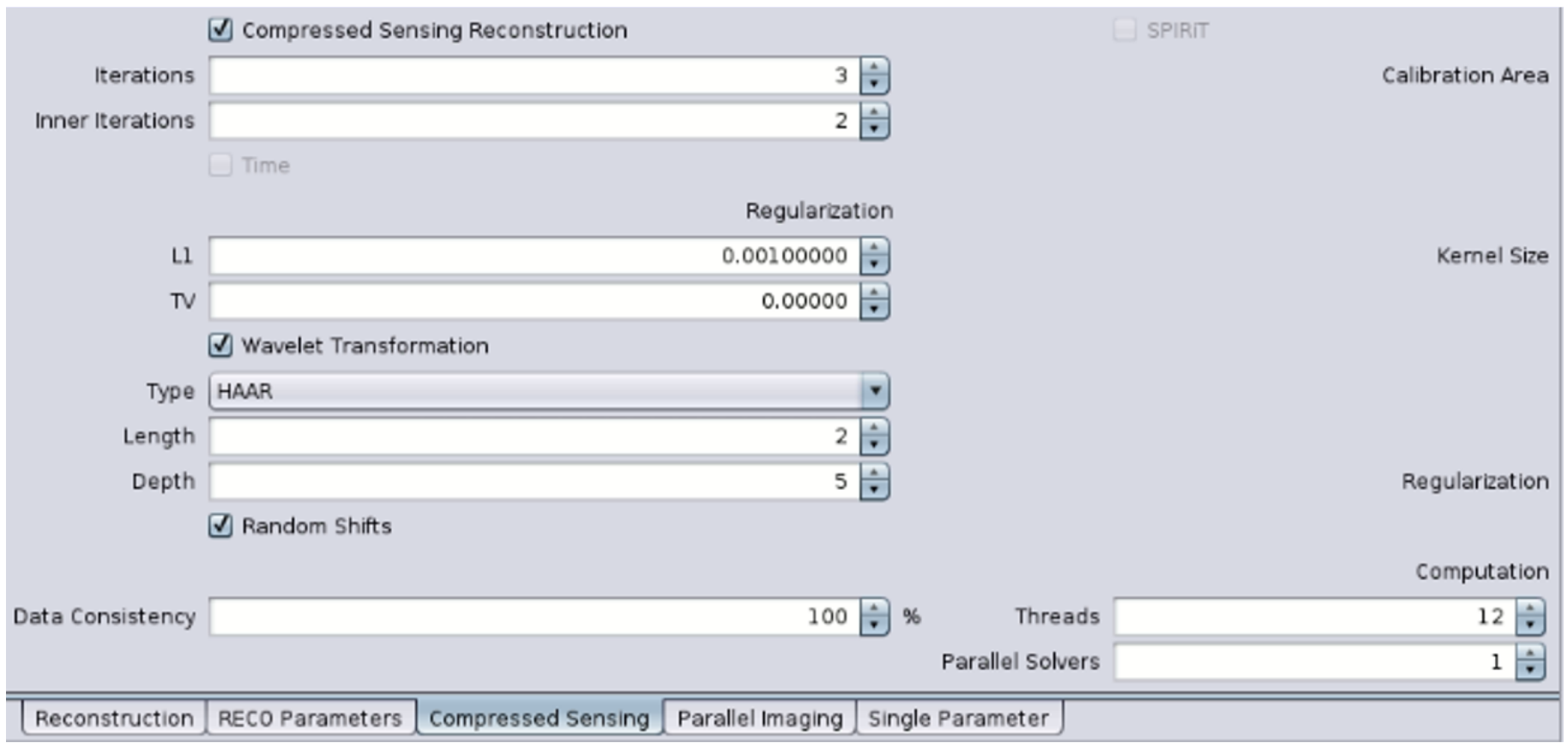

Figure S2: Compressed Sensing reconstruction parameters on the ParaVision interface.

3.2 *k*-space representation for CS

To visualize the k-space distribution of CS sampling, we simulated k-space with different CS parameters using a Shepp-Logan phantom,[S2] which consists of multiple elliptical structures with varying intensities, thereby simulating brain-like tissue contrasts (Figure S3). To mimic real MRI acquisition, Cartesian undersampling masks were simulated with a fully sampled central k-space area (CA) and uniform random sampling across the remaining k-space. Three CA values (100%, 43% and 22%) were compared at a fixed overall sampling fraction of 43%.

Overall, our simulated CS undersampling patterns demonstrated that preserving image quality is closely tied to the chosen sampling strategy, with particular emphasis on the importance of the central k-space for diagnostic accuracy.

3.3 Python code for CS simulation

A Python script was used to generate the Cartesian undersampling masks with given center area (CA) and overall sampling (S) parameters. In this convention, the total sampling fraction is $S\%$ of all k-space points, of which $CA\%$ are placed in a fully sampled central square and the remainder distributed uniformly at random. The code is attached as in PDF file.

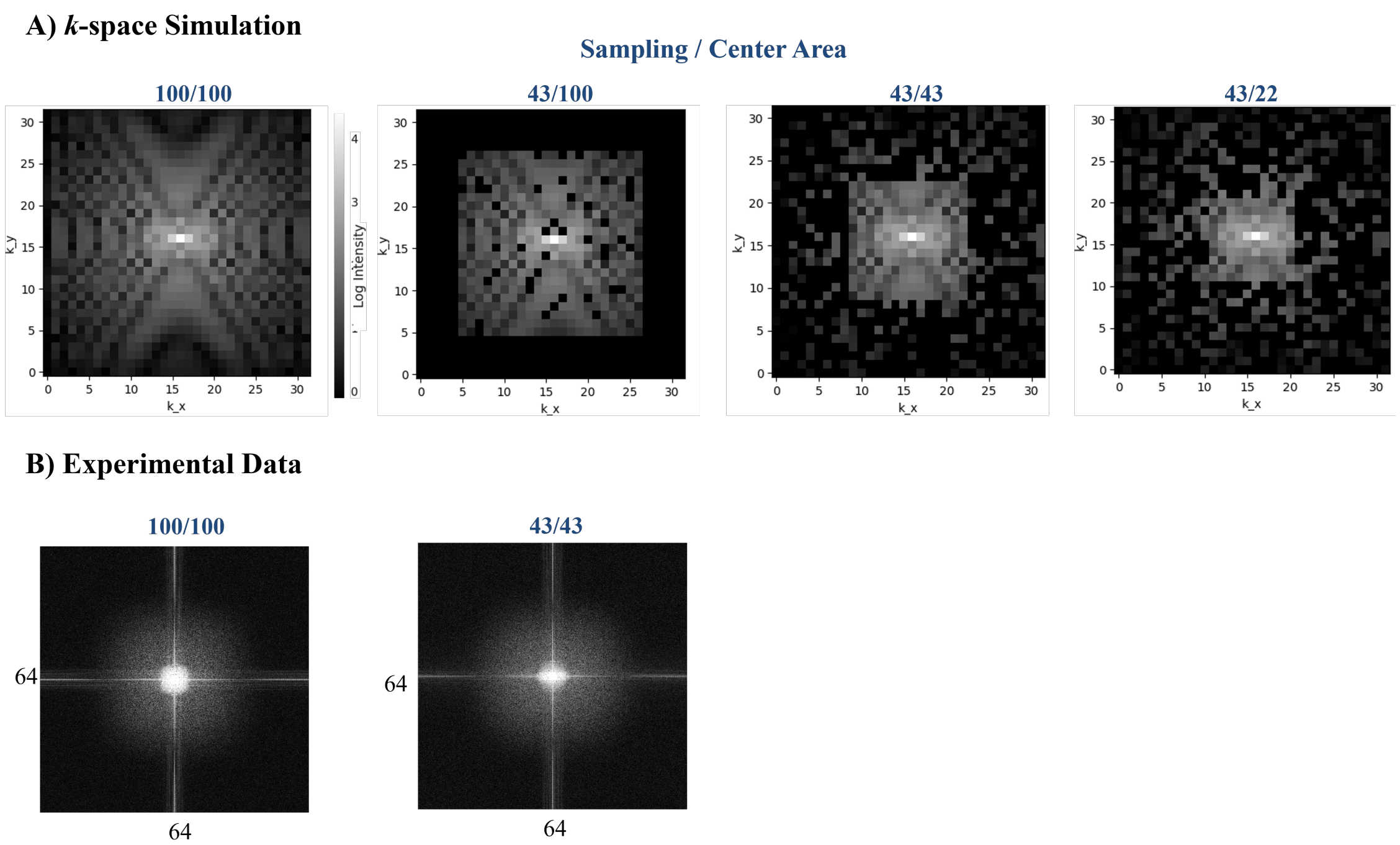

Figure S3: Simulated MRI k-space representation of Shepp-Logan phantom with varying degrees of compressed sensing undersampling and center k-space retention and experimental k-space data of the honeycomb image from Figure 3 in fully sampled and undersampled versions. Signal intensity is shown as the logarithmically scaled magnitude of the Fourier transform after applying the undersampling mask. The size of the images was MTX 32×32. The simulated image is for illustrative purposes and not derived from experimental data. The imaging parameters for the experimental data are given in the referred figure.

3.4 TFBA concentration optimization

To reliably detect displacement across a broad range of competitor binding affinities (K_C), the equilibrium between protein, reporter, and competitor must be tuned. In our previous setup, the TFBA concentration was 25 mM while the trypsin concentration was much lower. Under these conditions, the majority of TFBA molecules remained free in solution, and only a small fraction was bound to trypsin.

In the present study, without changing the trypsin concentration, we reduced the TFBA concentration to 5 mM. According to Equation 4 (please confirm reference), this adjustment theoretically increases the fraction of TFBA bound to trypsin and thereby enhances the detectable alterations upon competitor displacement of the bound TFBA. Based on these calculations, the detectable competitor affinity range (K_C) shifts from approximately 0.3 mM to 1.6 mM by lowering the reporter ligand concentration. Thus, the equation predicts that reducing TFBA expands sensitivity to weaker competitors. However, since the goal of this project is to maximize measurement acceleration, we did not further reduce TFBA concentration, as this would compromise SNR. For demonstration, we fitted the estimated K_C values as a function of TFBA concentration while keeping the protein concentration constant (Figure S4).

In particular, this adjustment improved sensitivity to weak competitors with higher K_C (Chapter **Binding affinity determination of lead competitors**), which would otherwise fail to produce measurable displacement at excess reporter concentrations.

3.5 Binding affinity classification for screening

Hits were classified according to their experimentally measured fractional displacement values ($F\%$), which were obtained from the dose-dependent R_2 changes in the competitive ^{19}F-MRI assay. Instead of comparing the competitor binding constant K_C directly to the reporter ligand's dissociation constant ($K_D^{\mathrm{TFBA}} = 142\,\mu\mathrm{M}$), the classification scheme relies on the empirically established correspondence between the observed displacement range and the

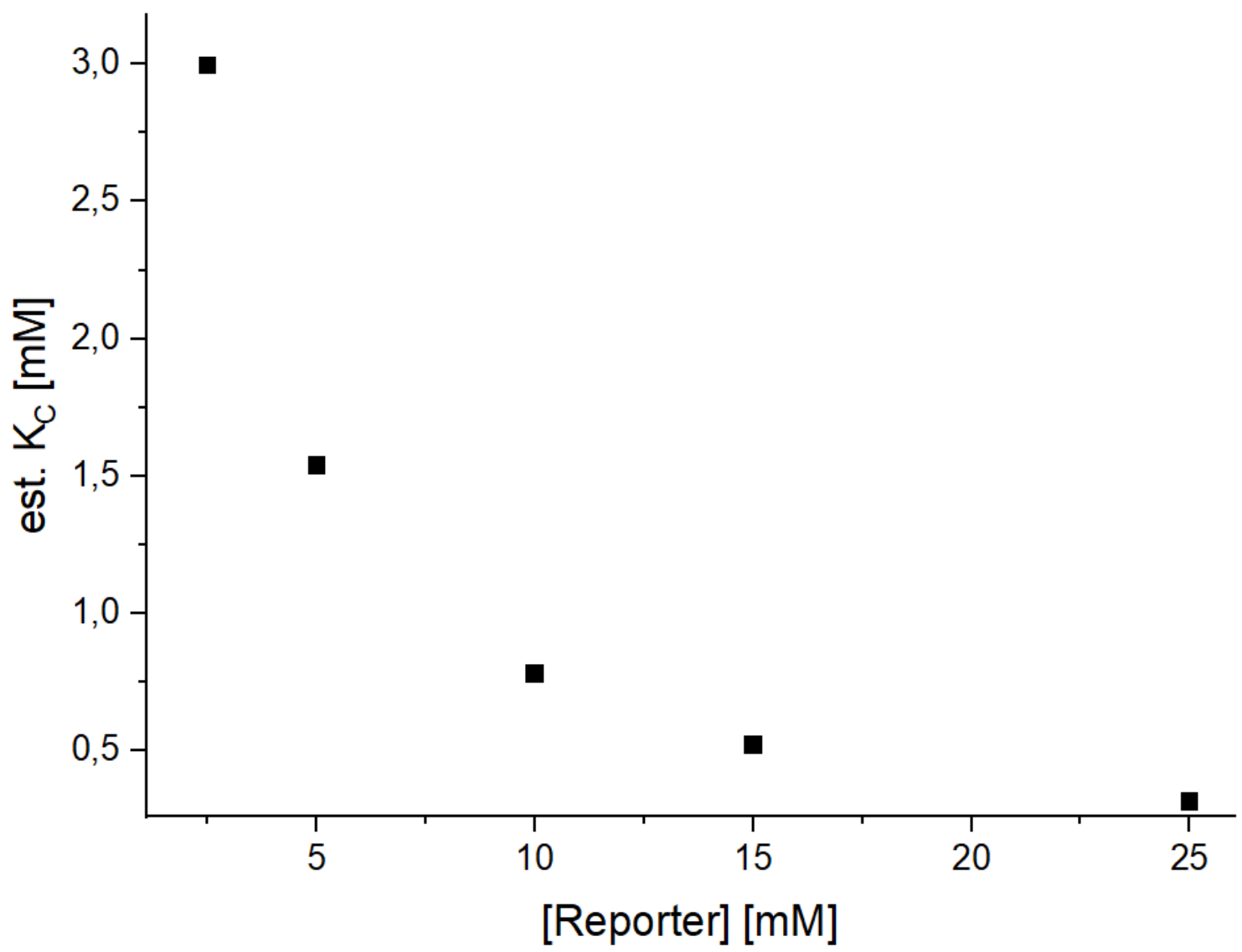

Figure S4: Estimated K_C as a function of reporter ligand concentration

expected K_C regime, as summarized in Table S3.

The numerical criteria used in this study were:

- **Strong:** $F = 99{-}85\%$ corresponding to $K_C = 8{-}140$ µM
- **Weak:** $F = 84{-}65\%$ corresponding to $K_C = 141{-}450$ µM
- **Very-weak:** $F = 64{-}30\%$ corresponding to $K_C = 451{-}2000$ µM

Thus, the regime assignment is derived directly from the displacement values. For example, a competitor exhibiting $F = 92\%$ is classified as *strong*, whereas one giving $F \approx 70\%$ is considered *weak*, and one with $F < 50\%$ falls into the *very-weak* regime.

3.6 PPAT - hit 10 R_2-relaxation rate

Binding and displacement were assessed by analysing both the dose-dependent changes in the reporter-ligand relaxation rate (R_2) and the quality of the corresponding regression fits. Only compounds that exhibited (i) a systematic concentration-dependent trend in R_2 or displacement, and (ii) a sufficiently high goodness of fit (R^2) were classified as true hits. Compounds failing to meet either criterion were excluded.

In the PPAT–TTAPC study, hit **10** showed random, non-monotonic scatter without any meaningful correlation, resulting in poor fit quality and indicating the absence of competitive displacement. In contrast, hit **15** displayed a clear dose-dependent response accompanied by a high R^2 value, supporting its identification as a true competitor (Figure S5).

A control experiment with increased amounts of hit 10 in DMSO demonstrated visible phase separation, further explaining the inconsistent relaxation behaviour (Figure S6).

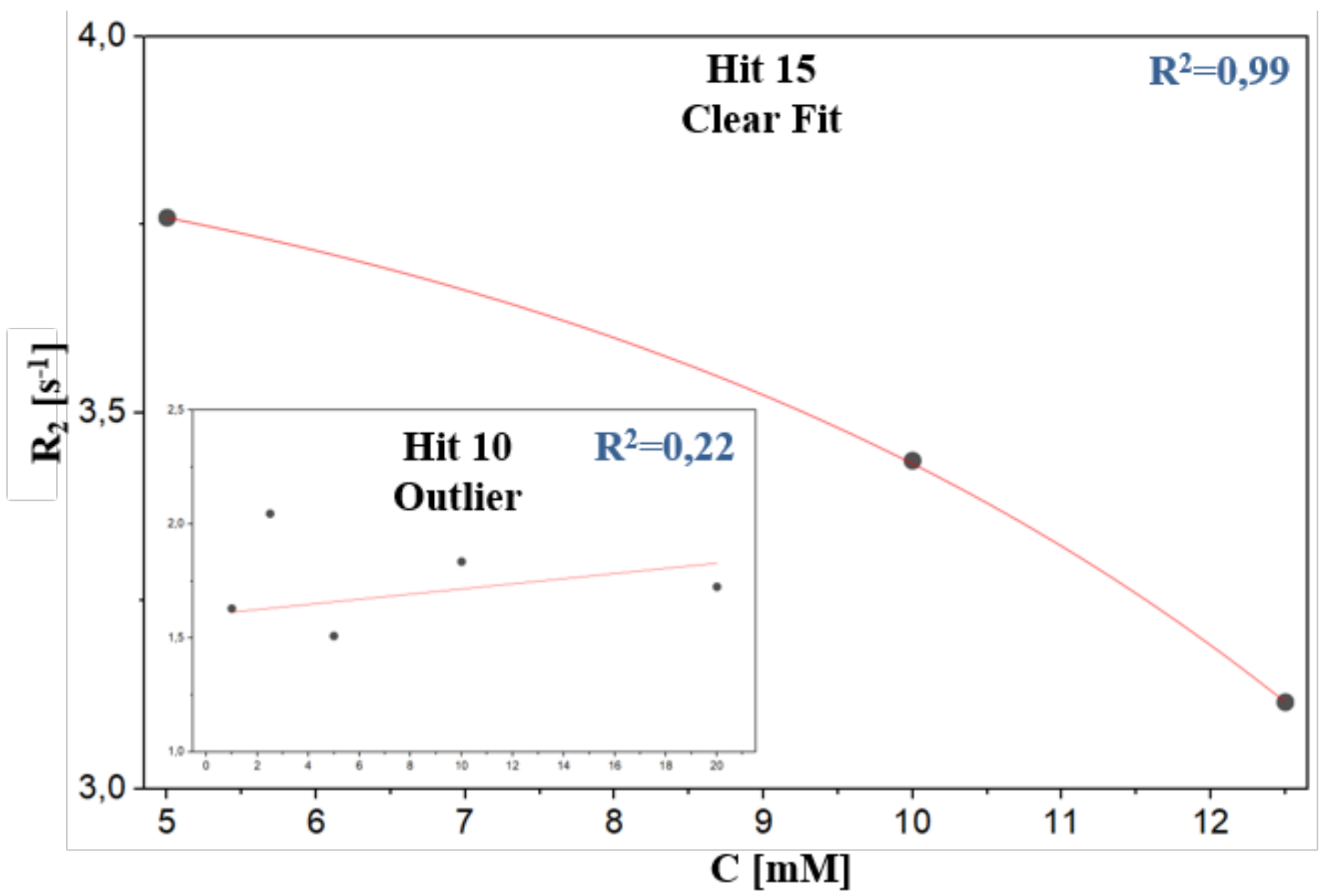

Figure S5: Dose-dependent relaxation rate alterations in comparison of a nonbinder hit 10 and binder hit 15 for the PPAT-TTAPC study. Black dots denote experimental data, and red lines the fitted models. The statistical coefficient of determination R^2 (blue) is given in each panel to indicate the quality of fit.

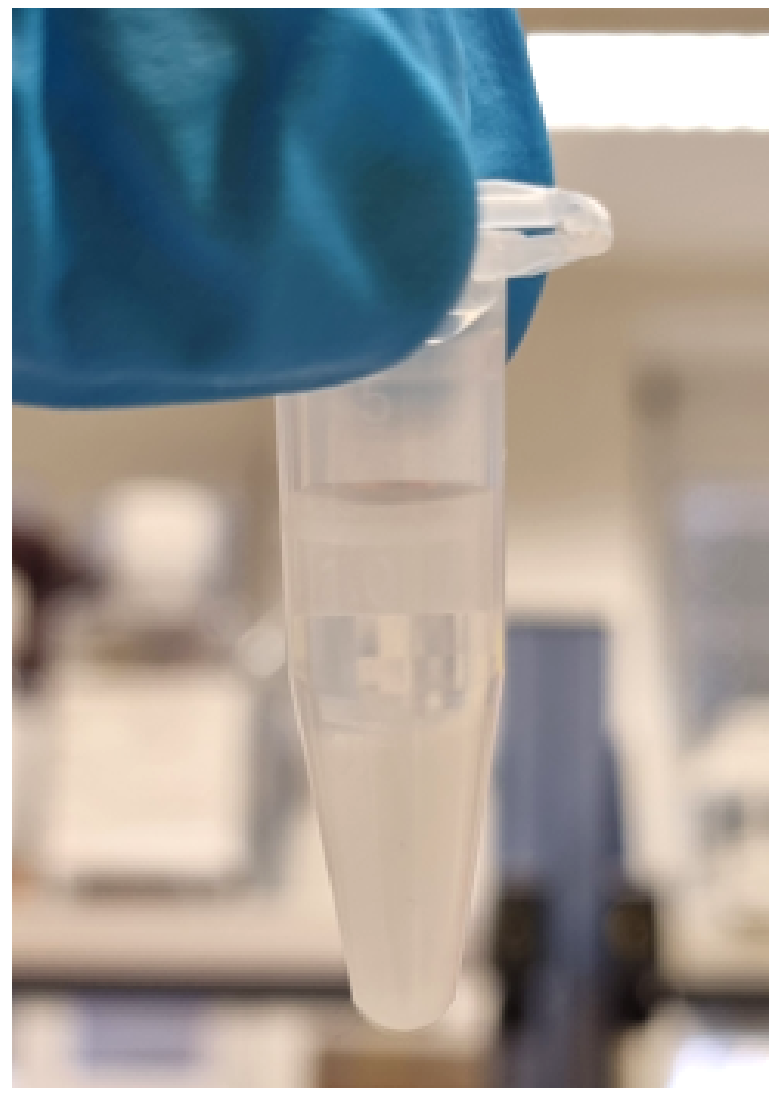

Figure S6: Phase separation of hit 10 upon DMSO addition as a control experiment. The sample separated into two visible phases, demonstrating limited compatibility of hit 10 with DMSO and preventing the formation of a homogeneous competitor solution.

Table S1: Hydrophobic compounds for drug screening

Solvent	Compound
DMSO	Aspartate
	Cystine
	Tyrosine
	Biphely-phenyl urea
	Benzoaminomethylphenol
	Methotrexate
	Aniline
	Salicylic acid
	Ibuprofen
Chloroform	Ergosterol
	Adamantane

Table S2: Hydrophilic compounds for drug screening

L-Alanine	Sodium acetate	Trizma acetate
R-2-Aminobutanol	S-2-Aminobutanol	Arginine
Asparagine	Cysteine	Methylbenzylamine
Glutamine	Aspartic acid	Glutamic acid
Glycine	Histidine	Isoleucine
Leucine	Lysine	Methionine
Phenylalanine	Proline	Serine
Threonine	Tryptophan	Valine
Glucose	Lactose	Sucrose
Benzamidine	Benzylamine	Starch
Benzoylarginineethylester	Imidazole	Hydroquinone
Glucosamine	L-Ascorbic acid	Glucono-delta-lactone
N-Hydroxysuccinimide	D-Maltose	Nicotinamide
Pyruvic acid	Alginic acid	Polyethylenglycol
D-Alanine	Hydroxy-Methylbutyric acid	Citric acid
Dimethylaminopropyl-Ethylcarboimide	Poly(vinyl alcohol)	Urea
Acetyl-L-phenylalanine	Mannitol	

Table S3: Comparison of the four ^{19}F-MRI T_2-mapping methodologies.

Methodology	Parallelization	[TFBA] [mM]	Volume [µl]	TA per sample [min]
Conventional	1	25	500	18.0
Capillary	9	25	200	6.0
Honeycomb	61	5	50	4.2
Honeycomb with CS (43%)	61	5	50	2.0

Table S4: Displacement of trypsin-competitors and their expected binding affinity regimes for the first step of screening classification

F %	K_C in µM	Regime
99 - 85	8 - 140	Strong
84 - 65	141 - 450	Weak
64 - 30	451 - 2000	Very-Weak

Table S5: Displacement and expected binding affinity regimes for TTAPC with PPAT enzyme

F %	Regime
99 - 85	Strong
84 - 65	Weak
64 - 30	Very-Weak

References

(S1) Faderl, D.; Chenakkara, A.; Jouda, M.; MacKinnon, N.; Gossert, A. D.; Korvink, J. G. Accelerated Screening of Protein–Ligand Interactions via Parallel T2-Weighted 19F-MRI. *Analytical Chemistry* **2024**, *96*, 9859–9865, PMID: 38830623.

(S2) Shepp, L. A.; Logan, B. F. The Fourier reconstruction of a head section. *IEEE Transactions on Nuclear Science* **1974**, *21*, 21–43.
